# Causes of acromion and scapular spine fractures following reverse shoulder arthroplasty: a retrospective analysis and literature review

**DOI:** 10.1007/s00264-020-04813-5

**Published:** 2020-09-29

**Authors:** Richard W. Nyffeler, Bartu Altioklar, Philipp Bissig

**Affiliations:** Orthopädie Sonnenhof, Buchserstrasse 4, 3006 Bern, Switzerland

**Keywords:** Acromion fracture, Scapula fracture, Stress fracture, Reverse shoulder prosthesis, Rotator cuff tear

## Abstract

**Purpose:**

Fractures of the acromion and the scapular spine are serious complications after reverse total shoulder arthroplasty. They concern about 4 to 5% of the patients and always result in a significant deterioration of shoulder function. Different causes have been taken into consideration, particularly stress or fatigue fractures. The purpose of the present study was to analyse our own cases and to discuss the causes reported in the literature.

**Methods:**

We reviewed our shoulder arthroplasty registry and the consultation reports of the last ten years. The charts and radiographs of all patients who had a post-operative fracture of the acromion or the scapular spine were carefully examined and the results were compared with those of an age- and gender-matched control group.

**Results:**

Twelve patients with an average age of 79 years sustained a fracture of the acromion (*n* = 6) or the scapular spine (*n* = 6). The time interval between the operation and the fracture averaged 26 months and ranged from three weeks to 70 months. Eight patients (67%) had a trauma. Seven of them reported a fall on the corresponding shoulder and one a heavy blow on the acromion. The four non-traumatic fractures were attributed to poor bone quality. All 12 patients had immediate pain and difficulty to actively elevate the affected arm. The time interval between the fracture and its diagnosis averaged ten weeks (0 to 10 months). At final follow-up, all patients could reach their face and refused further surgery. Two patients rated their result as good, six as acceptable and four as poor.

**Conclusions:**

Our study cannot support the hypothesis that most acromion and scapular spine fractures after RSA are the result of increased tension in the deltoid or stress fractures. In our series, the majority of the fractures were related to a fall. Implantation of a reverse prosthesis exposes the acromion and makes it more vulnerable to direct trauma. Non-traumatic fractures were associated with poor bone quality.

## Introduction

Reverse shoulder arthroplasty (RSA) has become a standard procedure for the treatment of elderly patients with pain and severe functional impairment of the shoulder. The short- and mid-term results are good [1, 2]; the rate of peri- and postoperative problems and complications, however, is relatively high [3–6]. They include limited range of motion [7–9], scapular notching [10, 11], instability [12, 13], component loosening [3, 14, 15] and fractures of the acromion and scapular spine [16–29].

The cause of these fractures is still controversial. Some authors consider them as stress fractures [16, 17, 22–24, 26] or bone injuries associated with osteoporosis [19, 21, 29], acromioclavicular (AC) joint osteoarthritis [24] or a drill hole close to the scapular spine for the fixation of the glenoid base plate [18, 25]. Other authors believe that the design of the prosthesis [26, 30], the location of the centre of rotation [29, 31] or transection of the coracoacromial ligament plays a role [32]. In only a few cases, trauma was reported as a triggering factor [17, 18, 28]. Understanding these fractures is of utmost importance because their treatment is challenging and their outcome is often poor. Therefore, the purposes of the present study were to report our own cases of acromion and scapular spine fractures and to review the pathomechanisms described in the literature.

## Materials and methods

We reviewed our local arthroplasty database from April 2009 to December 2019 and identified all patients with a fracture of the acromion or the scapular spine after RSA (fracture group). The charts and radiographs of these patients were carefully examined and the following parameters were recorded: age, gender, patient history, initial diagnosis, previous interventions on the same shoulder, size of the rotator cuff tear, surgical approach, associated procedures such as acromioplasty and AC joint resection, size and position of the prosthetic components. Active range of motion, pain (no, mild to moderate, severe) and subjective result (very good, good, satisfactory, poor) were determined at the latest follow-up. An age- and gender-matched control group consisting of three times as many patients without a fracture after RSA was formed and assessed with the same parameters.

### Prosthesis

**Fig. 1:**
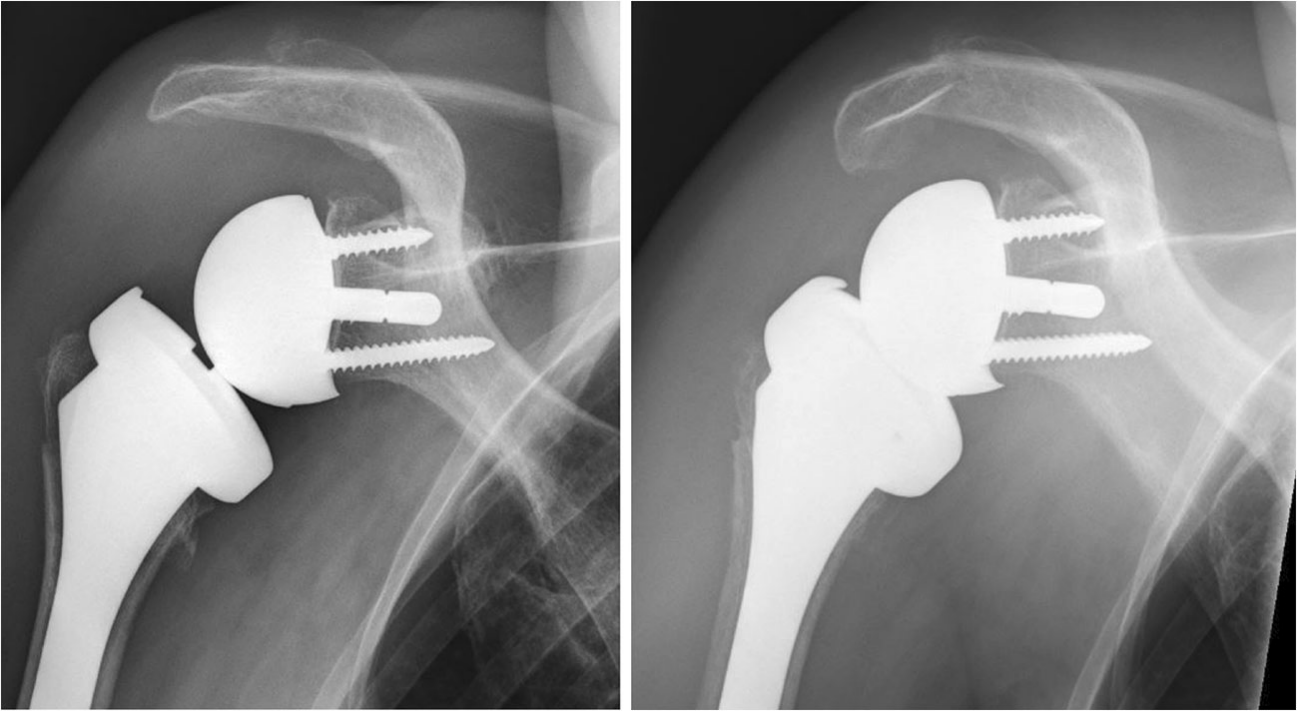
Anteroposterior radiographs of patient 4 treated with a reverse shoulder prosthesis for an acute humeral head fracture after a fall. The patient reported another accidental fall 3 month after surgery. X-ray pictures showed a type I acromion fracture

Since April 2009, the Duocentric prosthesis (Aston Medical, Saint-Etienne, France) was used in all cases treated in our clinic. It was designed with the intention to reduce the incidence of scapular notching and improving the range of motion. Its glenoid baseplate is characterized by an inferior extension, a 20-mm-long or 40-mm-long and 6-mm-thin central peg, and 3 screw holes for the fixation to the scapula. There are two sizes, for a 36-mm or 40-mm glenosphere. Its centre of rotation is lateral to the implant-bone interface. The humeral component includes an onlay design with a standard stem, an adjustable epiphyseal plate with a neck-shaft angle of 145°, and a symmetric liner of different thicknesses (4 mm, 9 mm and 12 mm). The prosthesis was designed for degenerative conditions and proximal humeral fractures (Fig. 1).

### Surgical technique and postoperative rehabilitation

All patients were operated under general anaesthesia. A deltopectoral approach was made when the subscapularis tendon was either partially or completely torn and in revision cases. A superolateral approach with detachment of the acromial insertion of the anterior deltoid was chosen when the subscapularis tendon was intact on pre-operative MRI or CT scan and in some fracture cases. The humeral head was resected at the level of the supraspinatus foot print, with either a specific guide or free hand. The labrum was completely excised to expose the superior and inferior glenoid contour. The glenoid baseplate was placed flush with the glenoid bone, at the level of the inferior glenoid rim. The superior fixation screw was oriented towards the coracoid process in order to minimize the risk of damaging the suprascapular nerve [10]. The humeral component was implanted with 0 to 20° of retroversion. The subscapularis was reattached with non-resorbable bone sutures, if detached and not retracted. Even the anterior deltoid was reattached with non-resorbable transosseous sutures. At the end of the procedure, the arm was immobilized in a sling with abduction pillow at 15° for six weeks, but early passive and active assisted shoulder mobilizations were allowed. Active use of the arm was encouraged after six weeks; lifting of light weights was allowed after three months. No formal strengthening exercises were prescribed.

### Radiographic evaluation

The fractures were classified into types I to III according to Levy et al. [33]. In this classification, type I fractures pass through the lateral border of the acromion, type II fractures through the posterior aspect of the acromion and type III fractures through the scapular spine, medial to the plane of the glenoid. Lengthening of the arm was determined on anteroposterior radiographs of the shoulder with the arm in adduction.

### Statistical analysis

Data analysis was performed according to the recommendations of Marusteri and Bacarea [34] and with use of the R software for statistical computing (R Foundation, Vienna, Austria). The Mann-Whitney *U* test was used for groups of unpaired data without normal distribution and the Kruskal-Wallis test was applied for categorial samples. Significance level was set at 0.05.

## Results

### Fracture group

**Fig. 2:**
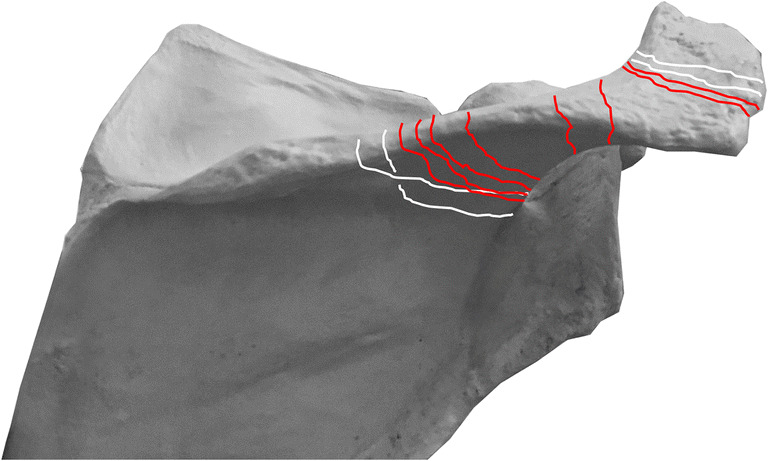
Graphic representation showing the fracture lines detected in our patient cohort. Type I fractures passed through the lateral border of the acromion, type II fractures through the posterior border of the acromion and type III fractures through the scapular spine. Traumatic fractures are represented with red lines, non-traumatic fractures with white lines

**Fig. 3:**
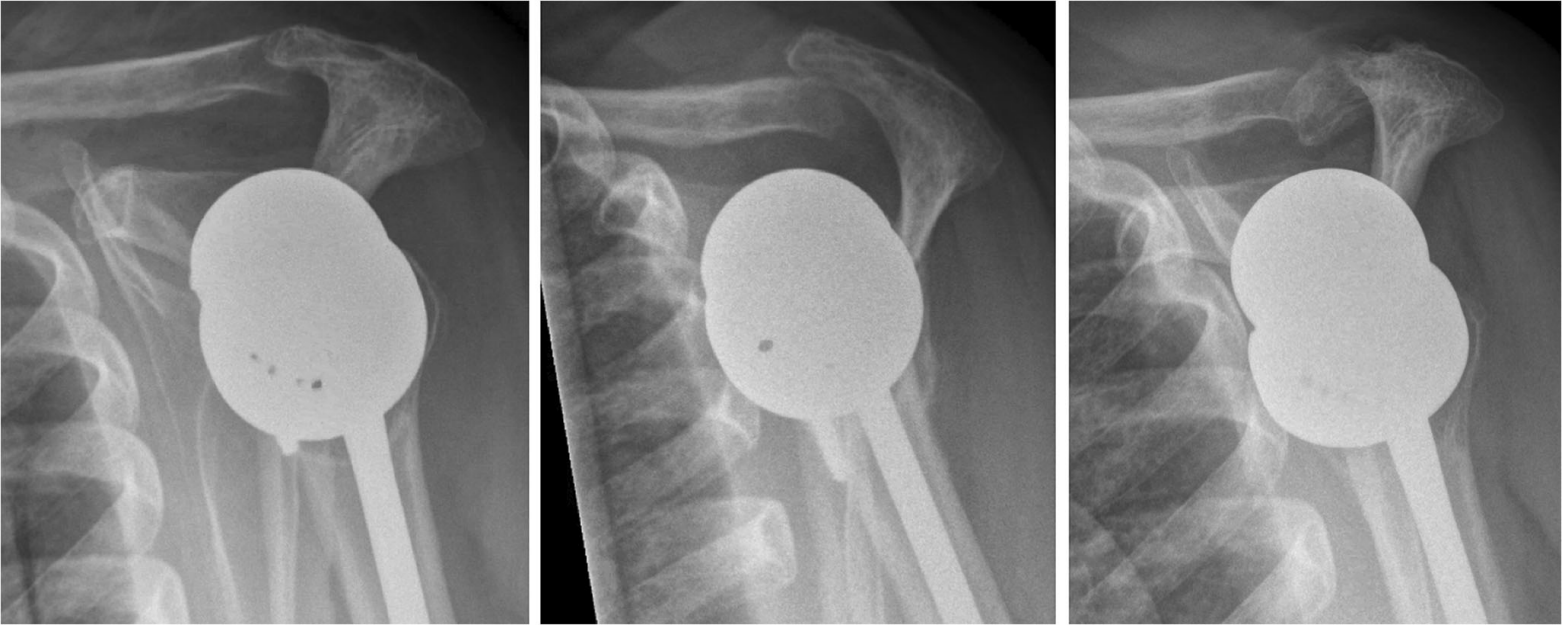
Lateral views of patient 2 showing a normal alignment of the lateral clavicle with the acromion 6 weeks after reverse shoulder arthroplasty, a dislocation of the clavicle under the acromion after 3 months and an acromion type I fracture after 4 months. The AC joint was resected during the procedure

**Fig. 4:**
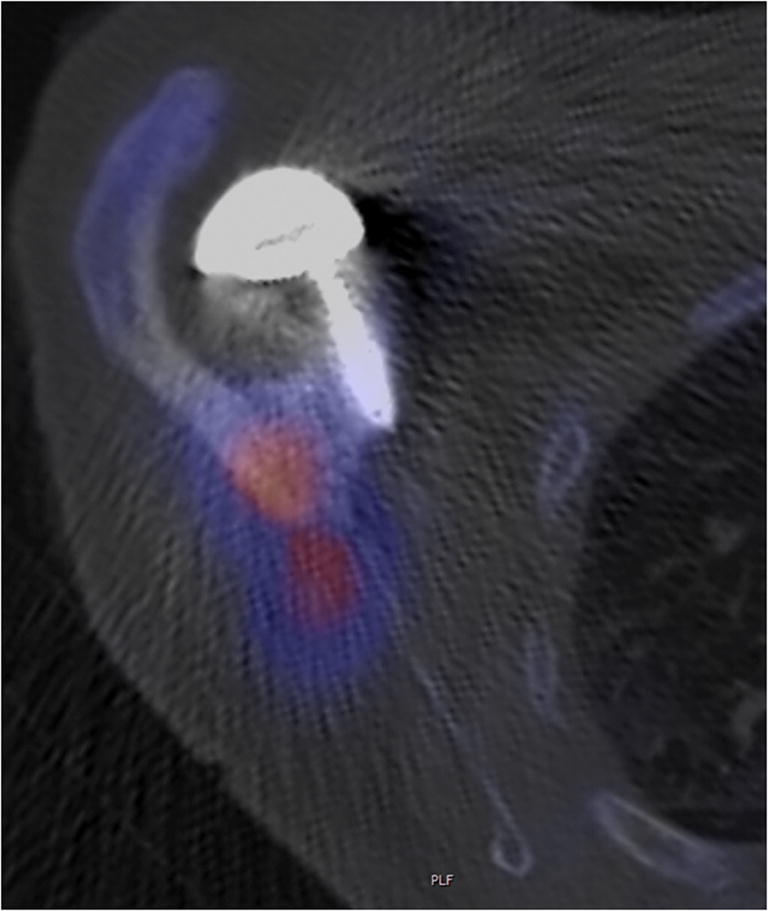
Axial single-photon emission computed tomography (SPECT) image of patient 11 demonstrates a scapular spine fracture on the right side with increased uptake (red area). The superior screw for the fixation of the glenoid base plate was not in the fracture zone

Eleven out of 189 patients (5.8%) treated in our clinic with a Duocentric prosthesis (Aston Medical, Saint-Etienne, France) and one patient operated in another hospital with an Affinis inverse prosthesis (Mathys Medical, Bettlach, Switzerland) and addressed for further controls were identified with a post-operative acromion (*n* = 6) or scapular spine (*n* = 6) fracture. The corresponding fracture lines are represented in Fig. 2 and the patient characteristics are listed in Table 1. The fractures occurred between three weeks and 70 months after replacement (average 26 months, median 13 months). The dates and the causes of the fractures were determined from patient history. Eight out of 12 patients (67%) reported a direct trauma to their shoulder. In seven cases, the fracture occurred after an accidental fall and in one case as a result of blunt trauma to the superior aspect of the shoulder caused by the head of a frightened horse. Of the four patients lacking trauma, one explained that the symptoms started during physical therapy (patient 1). The radiographs of another patient (patient 2) who had an additional AC resection during shoulder replacement surgery showed a normal alignment of the lateral clavicle with the acromion after six weeks, an inferior dislocation of the clavicle after three months and an acromion fracture after four months (Fig. 3). The third patient (patient 11) reported a vigorous arm movement behind the back 63 months after the operation. Standard radiographs were normal; however, a single-photon emission computed tomography (SPECT) revealed a fracture of the scapular spine (Fig. 4). The fourth patient devoid of trauma said that the symptoms started while she made housework (patient 12). All 12 patients reported a sudden onset of pain associated with a decrease of shoulder function. Eight patients saw a doctor within three days; the other four patients waited two, three, four and 39 weeks, respectively. In six cases, the fractures were diagnosed during the first consultation, and in the other six patients, the fractures were diagnosed with a delay of 15 to 90 days.

**Table 1:** Summary of the patient characteristics. *OA+cuff tear*, osteoarthritis with additional rotator cuff tear; *dp*, deltopectoral approach; *sl*, superolateral approach

Patient	Gender	Age at surgery	Side	Initial diagnosis*	Approach*	AC joint resection	Fracture type (Levy)	Circumstances	Interval RSA–fracture (months)	Interval fracture–first control (days)	Subjective result
1	m	81	Left	Massive cuff tear	sl	Yes	1	Physical therapy	3.0	1	Poor
2	f	74	Left	OA+cuff tear	dp	Yes	1	AC conflict	3.5	2	Acceptable
3	f	69	Right	Cuff tear arthropathy	dp	No	1	Heavy blow	18.5	26	Good
4	f	79	Right	Acute fracture	dp	No	1	Fall	3.0	21	Acceptable
5	f	85	Right	Cuff tear arthropathy	dp	No	2	Fall	0.7	0	Poor
6	f	80	Left	Dislocation + cuff tear	dp	No	2	Fall	5.8	15	Poor
7	m	88	Right	Cuff tear antropathy	sl	No	3	Fall	8.5	0	Good
8	f	69	Right	Cuff tear antropathy	dp	No	3	Fall	36.8	275	Acceptable
9	f	67	Left	Failed hemi	sl	Yes	3	Fall	47.4	3	Poor
10	f	80	Left	Failed cuff repair	sl	Yes	3	Fall	56.7	1	Acceptable
11	f	76	Right	Fracture sequelae	dp	No	3	Vigorous extension	62.9	0	Acceptable
12	f	68	Right	Cuff tear arthropathy	dp	No	3	Housework	70.1	0	Acceptable

The post-operative radiographs showed no malposition of the prosthetic components. The superior screw of the glenoid base plate projected onto the scapular spine in eight cases, but only in one case the rim of the scapular spine fracture passed close to the tip of the screw. All other fractures were superiorly and or laterally. CT scans of five patients revealed that the screws, which appeared to be in contact with the scapular spine on standard AP views were in reality in front of or antero-superiorly to it (Fig. 4). Lengthening of the arm averaged 23 mm (range 19 to 36 mm). In all patients, the displacement of the fractured fragment increased from the first diagnosis to the latest follow-up, even in those patients who were treated with an abduction splint.

At the latest follow-up, all patients were able to reach their mouth with the affected hand. Active elevation in the scapular plane averaged 95° and ranged from 60 to 140°. Active external rotation with the arm at the side was 22° (range 0 to 45°). All patients could reach at least their buttock. Three patients were pain free, eight patients had mild to moderate pain and one patient reported severe pain despite CT-proven fusion of the angulated acromion fragment. Denervation of the suprascapular nerve, performed under sonographic control by an anaesthetist, finally relived pain in the latter case. Two patients rated their result as good, six as satisfactory and four as poor. Patients with no pain and better active elevation were more satisfied. All patients accepted their functional limitations and refused further surgery.

### Control group

The age- and gender-matched control group included 36 patients with an average age of 73.6 years (range 56 to 83 years) and a follow-up of 48.7 months (range 12 to 108 months). Statistical analysis showed no significant difference between the control group and the fracture group with regard to age, gender, initial diagnosis, size of rotator cuff tear, approach, arm lengthening, diameter of the glenosphere, thickness of the polyethylene liner, additional procedures such as AC joint resection, or latissimus dorsi/teres major transfer. Active range of motion and patient satisfaction, however, were significantly better in the control group than in the fracture group.

## Discussion

Fractures of the acromion and the scapular spine are uncommon in individuals with normal shoulders [35, 36] or anatomic prostheses but are frequently encountered in patients with reverse shoulder prostheses. The fracture rate after RSA averages 4 to 5% [37–39] and ranges from 0.5% [40] to 25% [21]. In our series, it attained 5.8%, which is higher than the 4.3% reported in another study with an onlay design prosthesis [26] but still within the range of other series.

Many authors believe that these fractures are the result of arm lengthening and increased tension in the deltoid muscle [16, 17, 23, 24, 26]. However, finite element studies [41], biomechanical experiments [31, 42] and several clinical studies [22, 43] disapprove this hypothesis. Terrier et al. [41], for instance, simulated a shoulder with both a reverse and an anatomic prosthesis and calculated the forces necessary to fully elevate the arm. They found that implantation of a reverse prostheses decreased the force in the deltoid muscle by 20% when all rotator cuff muscles were deficient, and by 12% when only the supraspinatus was missing. Similarly, Ackland et al. [42], who performed a cadaver experiment with a reverse prosthesis, reported that the forces in the deltoid during abduction and flexion were significantly lower in the prosthetic shoulder than in the native joint. The findings of Dubrow et al. and Zmistowski et al. also raise doubts about the increased deltoid tension theory, because in one study arm lengthening did not differ between the fracture group and the control group [22] and in the other study deltoid length changes were smaller in patients with postoperative acromion pathologies than in patients without such complications [43]. Another argument is that obese patients with heavy arms and higher forces in the deltoid muscle are not affected by acromion or scapular spine fractures more often than slender patients. Finally, fractures of the coracoid process are uncommon, despite significant lengthening of both of the muscles originating on it during implantation of a reverse shoulder prosthesis as well.

The term “stress fracture” has very often been used in the context of acromion and scapular spine fractures [16, 17, 22–24, 26, 43, 44]. Its use must be questioned because stress fractures correspond to fatigue fractures that normally occur when normal bone is repetitively loaded over a long period of time. If acromion and scapular spine fractures were stress fractures, one would expect that their incidence increases with the time elapsed after the operation. This is obviously not the case. Most reported fractures appear within the first year after surgery [22, 26, 28, 37]. Neyton et al. [28] described 13 scapular fractures that already occurred at a mean 3.3 months postoperatively and Dubrow et al. [22] reported on 14 acromial fractures that occurred at an average of 5.1 months after RSA.

**Fig. 5:**
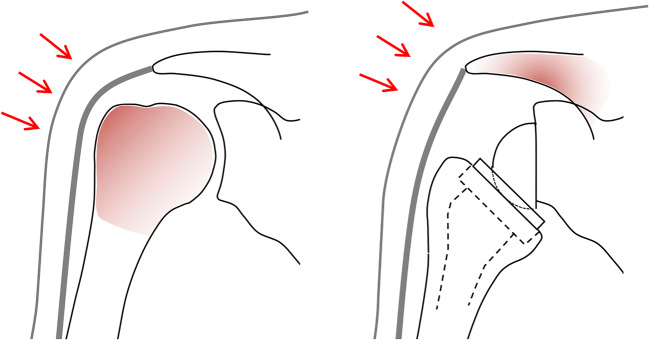
Schematic representation of a normal and a reconstructed shoulder. Implantation of a reverse prosthesis alters the shape of the shoulder. It exposes the acromion and makes it vulnerable to direct trauma. The red colour marks the area that absorbs the energy during a lateral impact

In previous studies, very few fractures have been associated with a traumatic event [17, 18, 28, 29]. Lau and Large [38] identified only 14 cases in their systematic review including 25 papers and 208 fractures. In contrast, a trauma was the principal cause for this complication in our cohort. Two factors may explain our findings: the risk of falling and the altered physiognomy. Elderly patients have a higher risk of falling, either due to balance disorders, decreased walking ability or visual impairment. All of our patients in the fracture group had at least one documented fall prior to the index procedure, and in two patients, a fall causing a humeral head fracture or a fracture dislocation was the reason for RSA. A fall resulting in a humeral head fracture was also the reason for RSA in many other studies [45–49]. Implantation of a reverse prosthesis does not influence the risk of falling, but it changes the contour of the shoulder. Medialization and distalization of the humerus expose the acromion and therefore make it more vulnerable to fractures. One could assume that the lateral impacts that occur after a simple fall are no longer absorbed by the humeral head but by the more prominent acromion (Fig. 5). Patients who fall with a reverse shoulder prosthesis may therefore sustain a fracture of the scapula, rather than a fracture of the proximal humerus. In our study, not all elderly patients spontaneously reported their fall during the planned follow-up control, but mentioned the trauma after being specifically asked about it. This may be related to trivialization of a fall by elderly patients and to pain subsidence within a couple of weeks. Zmistowski et al. [43] noted that over 10% of their patients were asymptomatic at the time of radiographic diagnosis of an acromion or scapular spine fracture. This could also explain why not more fractures have been associated with a trauma in the literature. We therefore recommend a careful interview focusing on the onset of symptoms, when a fracture of the acromion or the scapular spine is diagnosed during routine controls.

**Fig. 6:**
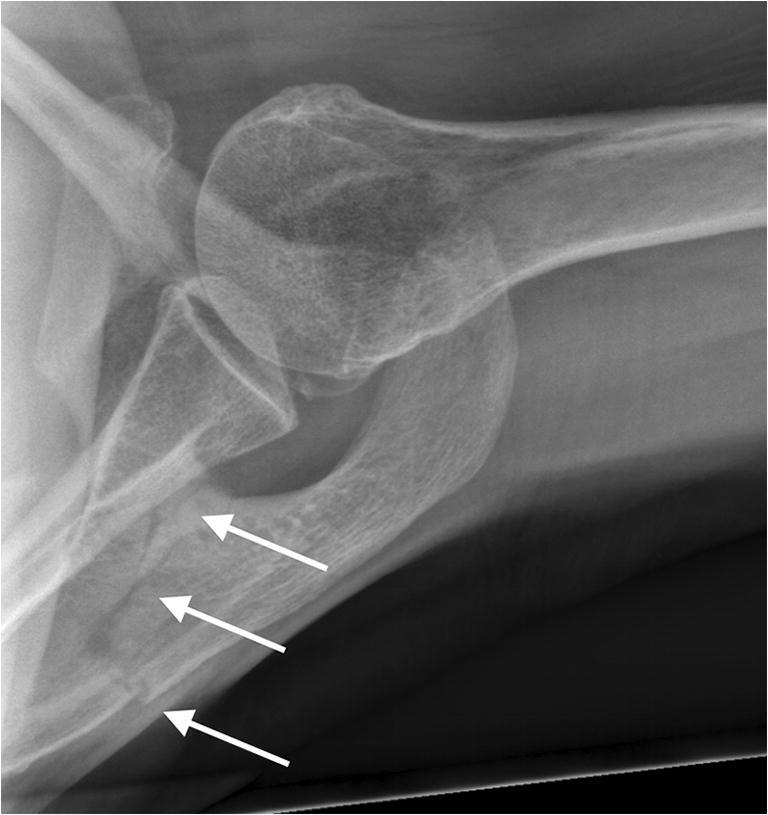
Axial view of the native left shoulder of patient 11 showing a non-traumatic fracture of the scapular spine

Four fractures in our series (33%) could not be associated with a fall. We assume that all of them were insufficiency fractures due to osteopenia or osteoporosis. We did not quantify the bone quality of the patients concerned; however, we looked for their comorbidities. Two of these four patients had sustained additional non-traumatic fractures of either their vertebral bodies, metatarsals or the opposite non-operated scapula (Fig. 6). Another patient was living with human immunodeficiency virus (HIV) which has been associated with a higher fracture risk [50, 51], and one patient was treated for osteoporosis. Osteoporosis has been considered a risk factor for acromion and scapular spine fractures after RSA in previous studies [21, 24, 27, 29, 52]. Besides general osteoporosis, local osteopenia might play a role. Protecting the shoulder and allowing only passive motion exercises during the first six weeks after the operation may unload and weaken the bone in such a way that it does not withstand the raising loads three to four months post-operatively.

Numerous other factors have been taken into consideration. Crosby et al. [18] described 3 scapular spine fractures that appeared to propagate from the tip of the superior screw for the fixation of the glenoid baseplate. The authors suggested that these screws acted as stress risers and they tried to confirm their hypothesis with a biomechanical experiment [25]. However, the experimental setup was designed to test the resistance of a glenoid component under a force directed from the humerus to the scapula. The deltoid muscle itself was not simulated in their experiments. Other authors [23] postulated that a stiff AC joint may cause a stress concentration in the acromion, resulting in a fracture after the patient has regained shoulder mobility. We could not confirm this hypothesis. In our series, resection of the AC joint was probably a cofactor for an acromion fracture (patient 2). Taylor and coworkers [32] introduced the scapular ring concept, which assumes that the coracoacromial ligament (CAL) can distribute strain patterns through the scapula. In their biomechanical experiment transection of the CAL during reverse shoulder arthroplasty resulted in increased strain at the scapular spine. The authors therefore suggested that preservation of the CAL could reduce the risk of scapular spine fractures. Because this ligament is routinely detached during a superolateral approach, one would expect a higher incidence of scapular spine fractures after a superolateral than after a deltopectoral approach. The review of Cho et al., however, showed that the opposite is the case [37].

**Fig. 7:**
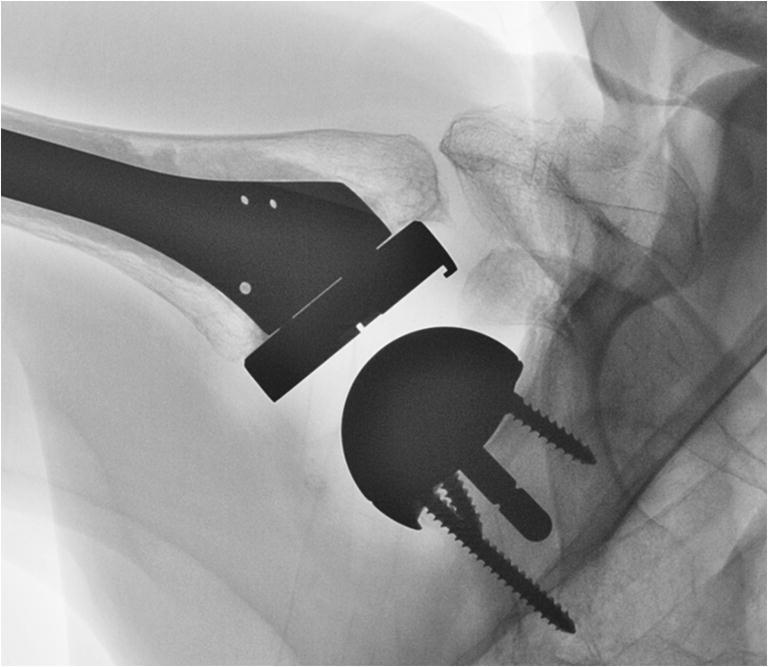
Anteroposterior radiograph of a Duocentric reverse prosthesis demonstrates a mechanical conflict between the greater tuberosity and the non-fractured acromion during abduction in the scapular plane

Other studies associated the position of the centre of rotation (COR) and the design of the reverse prosthesis with scapula fractures [26, 29, 44]. Schenk et al. [29] reported an increase of acromial fractures with high medialization and little distalization of the COR. Ascione et al. [26] found that COR lateralization with use of the bony increased offset technique (BIO-RSA) was not a significant predictor of scapular spine fractures. These authors however suggested that the higher incidence of postoperative fractures observed in their study could be related to the lateralized onlay stem. Implant configurations can indeed affect the lever arms and the forces in the deltoid muscle [31, 53] but these changes are small and should not influence the risk of scapula fractures. As noted above, implantation of a reverse prosthesis reduces the forces in the deltoid muscle. Accordingly, in a recent study with 2172 primary RSAs, Marigi et al. [54] could not find implant-related risk factors for scapula fractures, including medialized or lateralized COR or various glenosphere sizes. The higher rate of acromion fractures observed with onlay prostheses [26, 28] could possibly be related to an acromiohumeral conflict. Onlay prostheses lateralize the humerus to a greater extent than Grammont-style inlay prostheses, and as a result, the greater tuberosity makes bigger excursions and may hit against the acromion at lower abduction angles [31]. It is therefore conceivable that a fracture may be initiated during a forceful passive mobilization of the shoulder or during a vigorous active movement with the arm, especially when the bone is weak (Fig. 7). This pathomechanism could have played a role in two of our patients (patients 1 and 11).

**Fig. 8:**
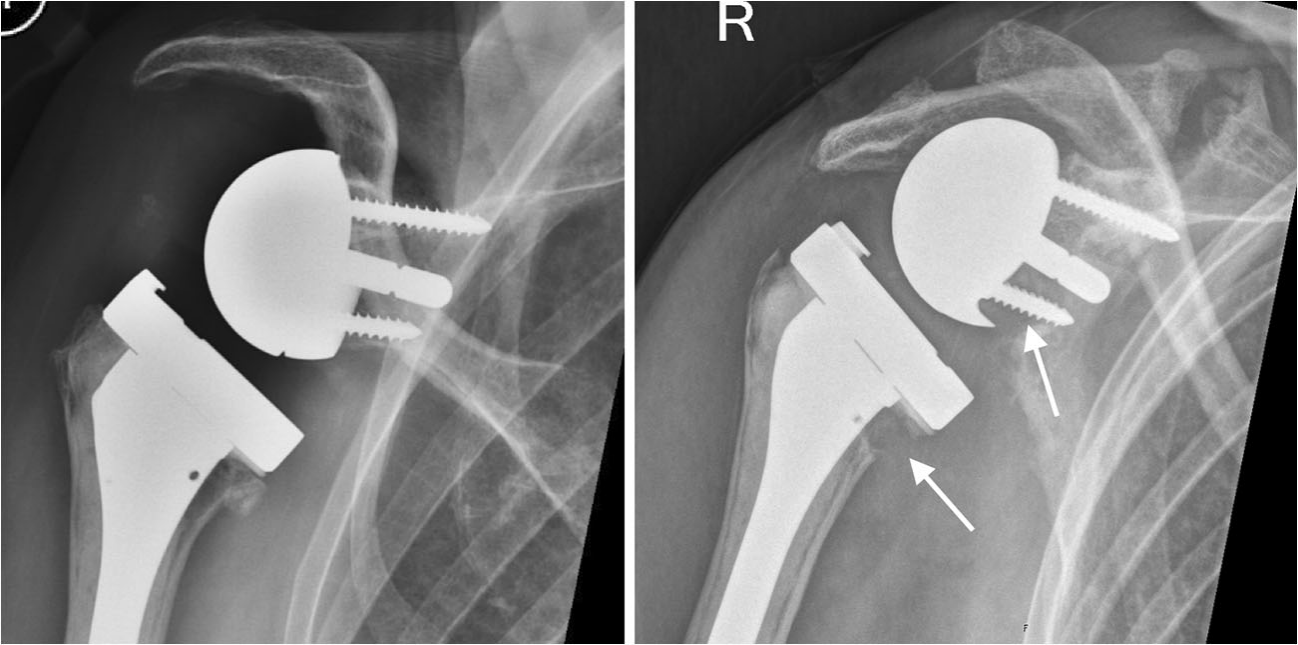
Anteroposterior radiographs of patient 8 showing an intact acromion 6 weeks postoperatively and a pseudarthrosis 18 months after a traumatic scapular spine fracture. Compensatory upward rotation of the scapula to avoid the subacromial conflict caused an inferior impingement with polyethylene wear, scapular notching and osteolysis of the calcar

**Fig. 9:**
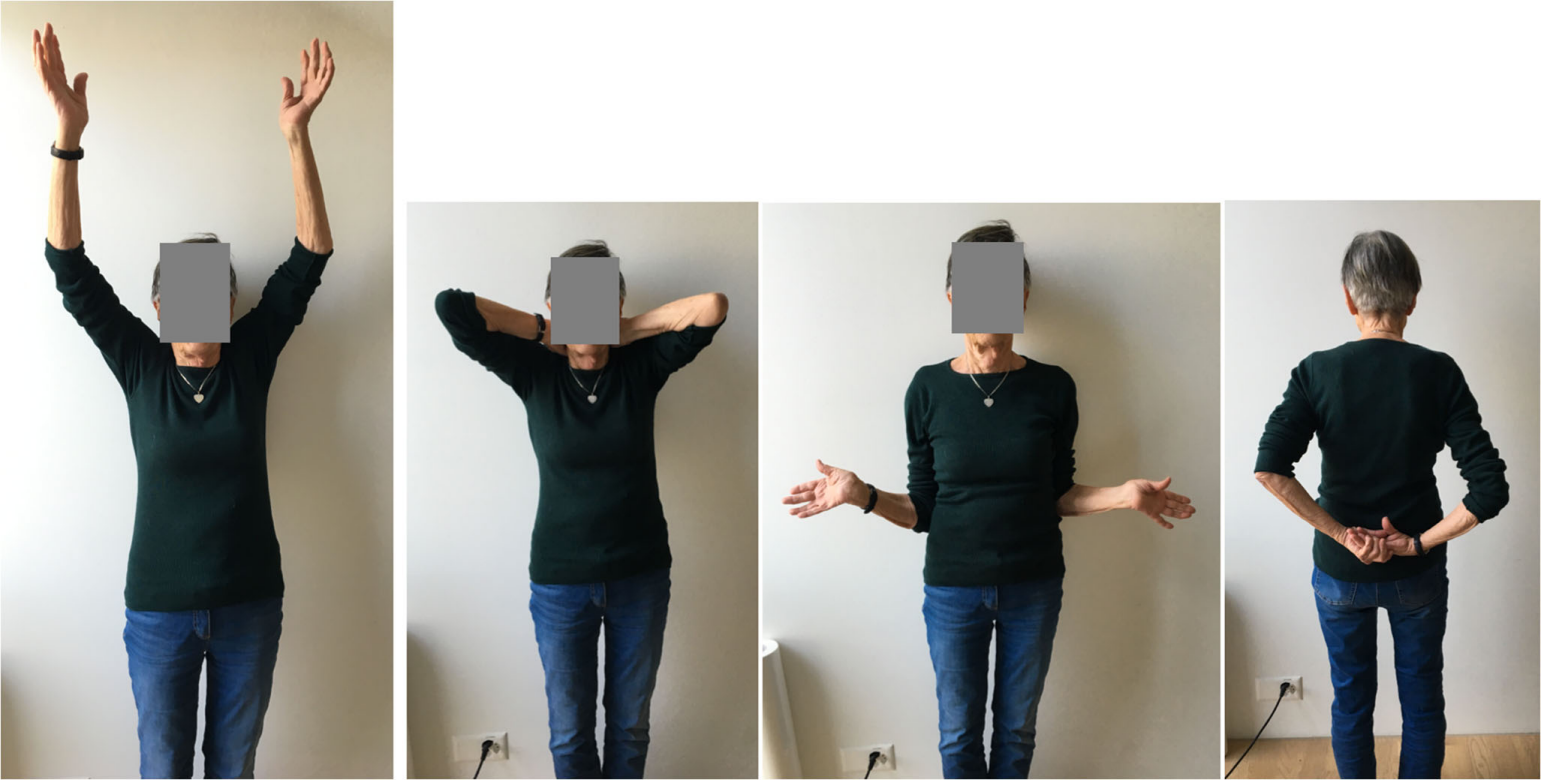
Functional result of patient 8 with the pseudarthrosis of the scapular spine on the right side

In our cohort, the functional results did not correlate well with the fracture type or the amount of displacement. Patient 8, for instance, had a good overhead shoulder function despite a pseudarthrosis of the scapular spine and a severely downward tilted acromion (Figs. 8 and 9). The compensatory upward rotation of the scapula, however, resulted in an inferior impingement with polyethylene wear, scapular notching and osteolysis of the calcar, an uncommon finding in patients treated with a Duocentric prosthesis.

This study has several limitations. First, it is a retrospective mono-centre study with a relatively small sample size. The data was prospectively collected, but the patients were not recalled for the purpose of this study. Accordingly, we cannot exclude that additional patients had sustained a fracture after the latest clinical and radiographic control in our practise and, as a result, the reported fracture rate might therefore be underestimated. Second, the follow-up after acromion or scapular spine fractures is short and, therefore, we cannot provide information about the long-term results of conservative treatment. However, the principal aims of our study were to point out the high percentage of traumatic fractures and to critically review the other pathomechanisms reported in the literature.

## Conclusions

Our study cannot support the hypothesis that most acromion and scapular spine fractures after RSA are the result of increased tension in the deltoid or stress fractures. In our series, the majority of fractures were related to a fall. Implantation of a reverse prosthesis exposes the acromion and makes it more vulnerable to direct trauma. Non-traumatic fractures were associated with poor bone quality.
